# Effect of osmolytes on the conformation and aggregation of some amyloid peptides: CD spectroscopic data

**DOI:** 10.1016/j.dib.2016.04.070

**Published:** 2016-05-04

**Authors:** Mohammed Inayathullah, Jayakumar Rajadas

**Affiliations:** a Biomaterials and Advanced Drug Delivery Laboratory, School of Medicine, Stanford University, Palo Alto, CA 94304, USA; b Bioorganic and Neurochemistry Laboratory, Central Leather Research Institute, Adyar, Chennai, Tamilnadu 600020, India; c Cardiovascular Pharmacology Division, Cardiovascular Institute, School of Medicine, Stanford University, Stanford, CA 94305, USA

**Keywords:** Amyloid β-protein, Aggregation, Circular dichroism, Conformation, Islet amyloid polypeptide, Osmolytes, Peptides, Polyglutamine

## Abstract

Protein misfolding and aggregation are responsible for a large number of diseases called protein conformational diseases or disorders that include Alzheimer׳s disease, Huntington׳s diseases, Prion related encephalopathies and type-II diabetes (http://dx.doi.org/10.1038/35041139) (Kopito and Ron, 2000) [1]. A variety of studies have shown that some small organic molecules, known as osmolytes have the ability to stabilize native conformation of proteins and prevent misfolding and aggregation (http://www.la-press.com/article.php?article_id=447) (Zhao et al., 2008) [2]. It has been shown that certain short segment or fragment of respective proteins can also form amyloids, and the segments also promote the aggregation in the full-length protein (http://dx.doi.org/10.2174/0929867023369187) (Gazit, 2002) [3]. This article presents circular dichroism spectroscopic data on conformational analysis and effect of osmolytes on Aβ peptide fragments, different lengths of polyglutamine peptide and the amyloidogenic segment of islet amyloid polypeptide.

**Specifications Table**

**Table:** 

Subject area	*Chemistry, Biology, Medicine*
More specific subject area	*Biophysical Chemistry*
Type of data	*Spectra, graph, figure*
How data was acquired	*Circular Dichroism* (*CD*) *spectroscopy* (*Jasco J-715 model spectropolarimeter*), *Infrared spectroscopy* (*Thermo Nicolet, AVATAR 320 Model*)
Data format	*Analyzed data*
Experimental factors	*The peptides were disaggregated using TFA/HFIP before starting the experiments*. *The peptides were dissolved and incubated in phosphate buffer or osmolyte solutions*.
Experimental features	*Secondary structure of peptides was determined. Time dependent conformational changes were measured. Effect of osmolytes on the aggregation was monitored*.
Data source location	*Chennai, India, and Stanford, USA*
Data accessibility	*The data is with this article*.

**Value of the data**

• Circular dichroism (CD) and infrared (IR) spectroscopic data of peptide fragments of amyloid β-protein (Aβ), poly-glutamine (polyQ) and islet amyloid polypeptide (IAPP) provides the secondary structure information in their self-assembled and/or aggregated states. This data is useful in understand conformational dynamics of the smaller peptide analogs of the full length amyloid proteins/peptides.
• Time-dependent CD data provide information on the aggregation kinetics and conformational transitions of the amyloid peptides on aging. In the present data the conformational transitions that occur during amyloid formation from a random coil to β-sheet, sometimes contains intermediate structures with helical or mixture of other conformations.
• Data on CD in presence of osmolytes show their influence on the aggregation of the amyloid peptides. This data can be useful for developing fibrilization inhibitor or promotor/accelerator for the amyloid peptides/proteins. The osmolytes that stabilize the protein/peptide conformation can be a potential candidate against protein conformational diseases [1], [2], [3].

## 1 Data

**Fig. 1:**
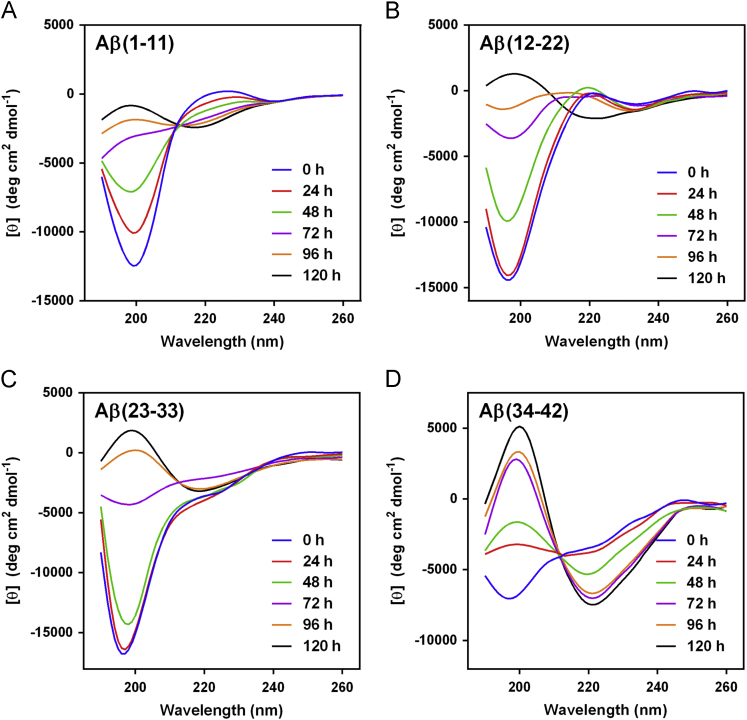
CD spectra of Aβ peptides (A) Aβ(1–11), (B) Aβ(12–22), (C) Aβ(23–33) and (D) Aβ(34–42) in 10 mM phosphate buffer, pH 7.4.

**Fig. 2:**
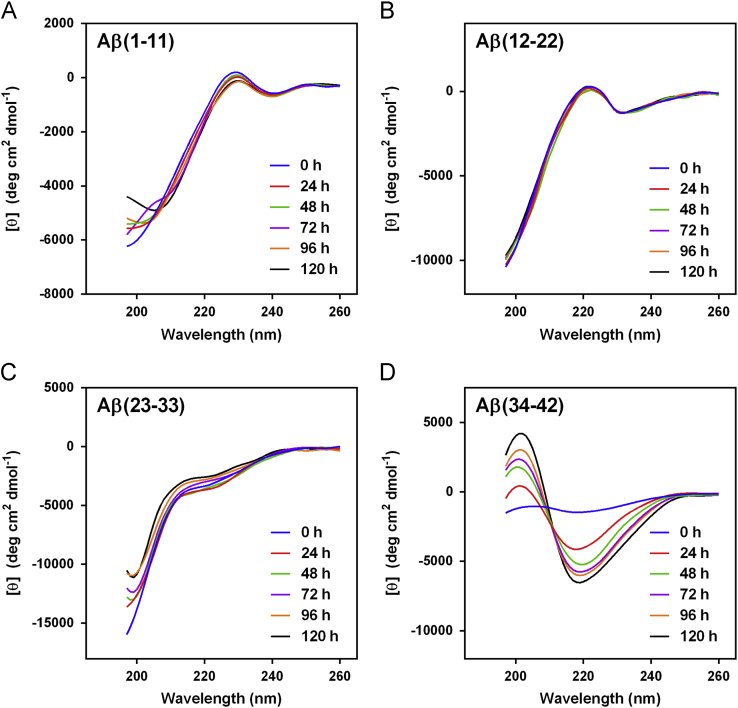
CD spectra of Aβ peptides (A) Aβ(1–11), (B) Aβ(12–22), (C) Aβ(23–33) and (D) Aβ(34–42) in 20%(v/v) glycerol.

**Fig. 3:**
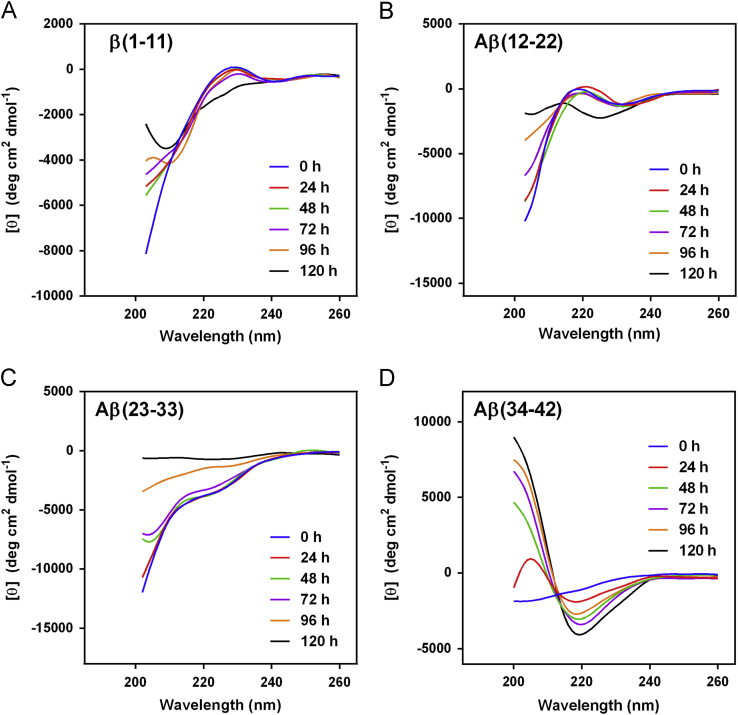
CD spectra of Aβ peptides (A) Aβ(1–11), (B) Aβ(12–22), (C) Aβ(23–33) and (D) Aβ(34–42) in 1 M TMAO.

**Fig. 4:**
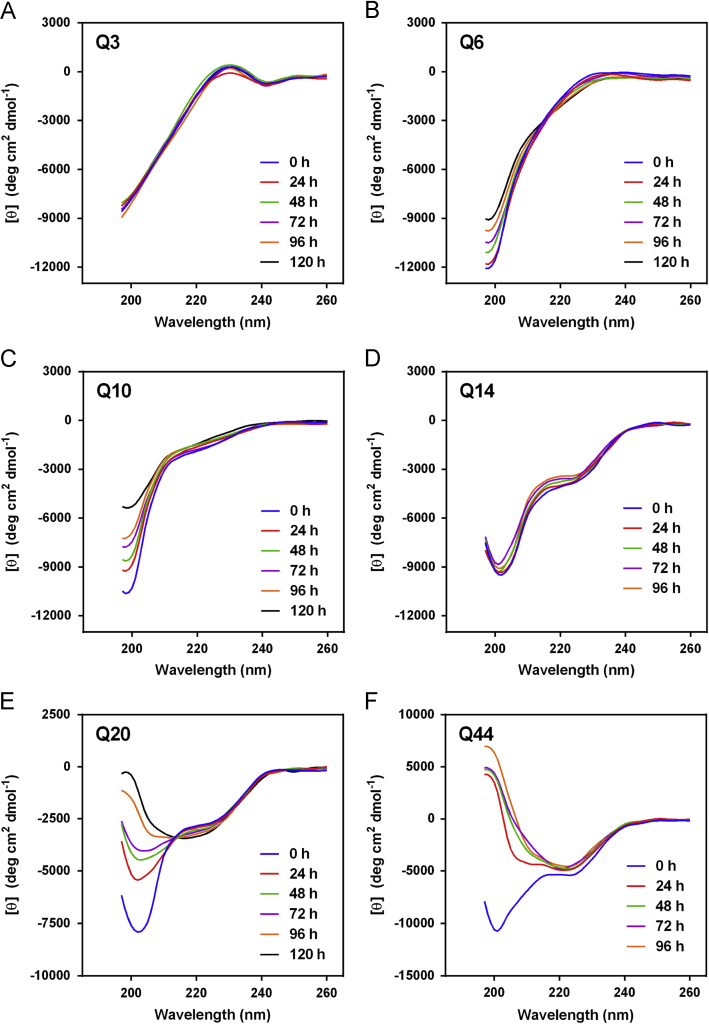
CD spectra of polyQ peptides (A) Q3, (B) Q6, (C) Q10, (D) Q14, (E) Q20 and (F) Q44 in 20%(v/v) glycerol.

**Fig. 5:**
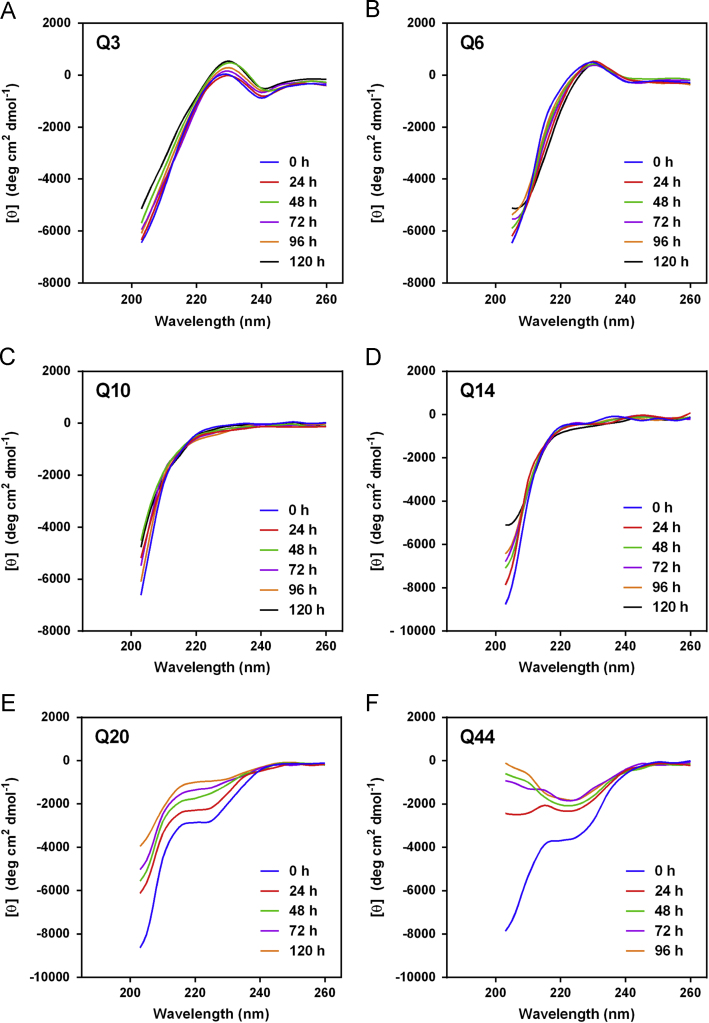
CD spectra of polyQ peptides (A) Q3, (B) Q6, (C) Q10, (D) Q14, (E) Q20 and (F) Q44 in 1 M TMAO.

**Fig. 6:**
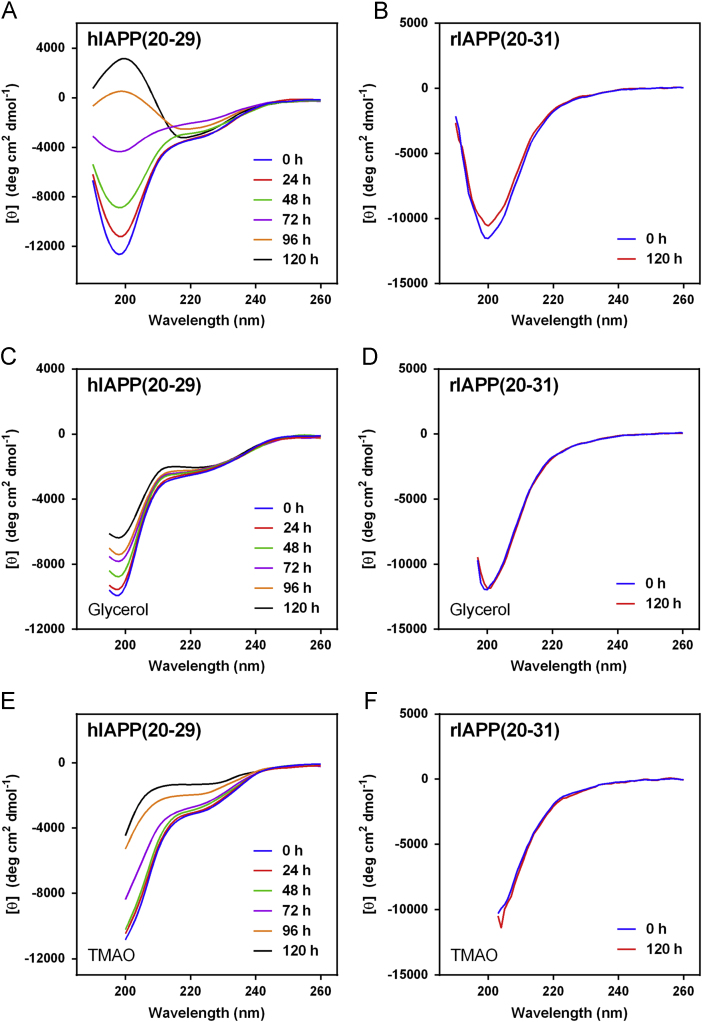
CD spectra of IAPP; hIAPP(20–29) (A, C and E) and rIAPP(20–31) (B, D and F), in 10 mM phosphate buffer pH 7.4 (A, B), in 20%(v/v) glycerol (C, D), and in 1 M TMAO (E, F).

The data presented here contain CD spectra of short peptide domains of Amyloid β-protein (Aβ) (Fig. 1, Fig. 2, Fig. 3), polyglutamine (polyQ) (Fig. 4, Fig. 5) and islet amyloid polypeptide (IAPP) (Fig. 6). Time dependent CD spectra of the peptides in presence of osmolytes have been presented. The CD data in absence of osmolytes presented here are peptide segments of Aβ and IAPP only. The data on polyQ without osmolytes have been published elsewhere [4]. IR spectra of some peptides without osmolytes have been shown in the supplemental data.

## 2 Experimental design, materials and methods

### 2.1 Experimental design

The specific domains or segments of the amyloidogenic proteins used here were: Aβ peptide fragments, Aβ(1–11), Aβ(12–22), Aβ(23–33) and Aβ(34–42); polyQ peptides: Q3, Q6, Q10, Q14, Q20 and Q44; IAPP peptides: hIAPP(20–29) and rIAPP(20–31) (negative control). We used CD spectroscopy to monitor the conformational changes that occur during the transition to β-sheet conformation that indicate amyloid formation at different time points. As osmolytes are known to prevent aggregation or amyloid formation, we used the osmolytes glycerol and TMAO to monitor those conformational changes. The time points chosen to record CD spectra were 0 h, 24, 48, 72, 96 and 120 h.

### 2.2 Peptide synthesis

Aβ peptide fragments: Aβ(1–11), Aβ(12–22), Aβ(23–33) and Aβ(34–42), polyQ peptides: Q3, Q6, Q10, Q14, Q20 and Q44, IAPP peptides: hIAPP(20–29) and rIAPP(20–31), were chemically synthesized, purified, and characterized as described [4], [5], [6]. Briefly, peptides were synthesized by solid phase peptide synthesis method using Fmoc (9-fluorenylmethoxy carbonyl) strategy. The purity of the peptides was >95%, analyzed using reverse-phase high-performance liquid chromatography and the molecular weight was determined by MALDI-TOF mass spectroscopy.

### 2.3 Sample preparation

The peptides were disaggregated using TFE/HFIP procedure [7]. The peptides were dissolved in 10 mM phosphate buffer pH7.4 or osmolyte solutions; 20% (v/v) glycerol or 1 M trimethylamine-N-oxide (TMAO) for CD measurements. Freshly prepared peptide solutions at a concentration of 0.3 mg/mL were used for the experiments.

### 2.4 Circular dichroism spectroscopy

Spectra were acquired periodically during incubation of the peptides at 25 °C. The peptide solution was placed into a 0.1 cm path length quartz cuvette. Spectra were acquired using a Jasco Model J-715 spectropolarimeter (Jasco, Japan) from ≈190 to 260 nm at 0.2 nm resolution with a scan rate of 100 nm/min. Five scans were acquired and averaged for each sample. Raw data were manipulated by smoothing and subtraction of buffer spectra according to the manufacturer׳s instructions. The data is represented in mean residue molar ellipticity [*θ*] (deg cm^2^ dmol^−^^1^). For some samples, high photomultiplier voltage at low wavelength precluded data acquisition to wavelengths as low as 190 nm.

The CD spectra of far-UV region (typically 190 to 240 nm) generally reflects the secondary structure content of the proteins and peptides [8]. The common secondary structure motifs such as the α-helix, β-pleated sheets, β-turns, and poly-L-proline II have very characteristic CD spectra [9]. A negative band near 218 nm and a positive band in the 195 to 200 nm region are characteristic of ß-sheet conformation. A negative band near 198 nm region is characteristic of random coil structure. α-helices are characterized by negative bands at 222 nm and 208 nm and a positive band between 190–195 nm.

### 2.5 Data processing

Data were processed and plotted using the graphical software “GraphPad Prism 6”.
