# Supplementary material for: Effect of osmolytes on the conformation and aggregation of some amyloid peptides: CD spectroscopic data
Source: Data Brief. 2016 May 4;7:1643–51. doi: 10.1016/j.dib.2016.04.070 (PMC4872718; doi:10.1016/j.dib.2016.04.070)

**Supplemental information**

**Effect of osmolytes on the conformation and aggregation of some amyloid peptides: CD spectroscopic data**

Mohammed Inayathullah and Jayakumar Rajadas

**Supplemental methods**

*Fourier Transform Infrared Spectroscopy*

Spectra were acquired using Thermo Nicolet (AVATAR 320 Model) at 25 °C. Spectra for solid samples were recorded using Attenuated Total Reflectance (ATR) method on a Ge (Germanium) crystal. Peptide solutions in D_2_O were measured using CaF_2_ cell (Thermo Nicolet) with a 0.05 mm Teflon spacer. For each spectrum 1000-scan interferograms were collected in the single-beam mode with a 2cm^-1^ resolution. The spectra were processed by OMNIC (version 6.0) software supplied by the manufacturer. Background/reference spectrum containing no sample was subtracted, and the spectra were smoothened and base line corrected. Fourier Self Deconvolution (FSD) and Peak fitting processes were performed using Thermo Galactic GRAMS-LT software for the amide I region of 1700-1600 cm^-1^.

**Supplemental Figure Captions**

**Supplementary Figure S1**. Solid state ATR-FTIR spectra of the amide-I region of the lyophilized peptides (A, D, G, J); FTIR absorption spectra of freshly dissolved peptide solutions (10 mg/ml, in D_2_O) (B, E, H, K); and aged (5 days) peptide solutions (C, F, I, L); of Aβ(1-11) (A, B, C), Aβ(12-22) (D, E, F), Aβ(23-33) (G, H, I) and Aβ(34-42) (J, K, L).

**Supplementary Figure S2.** Solid state ATR-FTIR spectra of the amide-I region of the lyophilized peptides (A, D); FTIR absorption spectra of freshly dissolved peptide solutions (10 mg/ml, in D_2_O) (B, E); and aged (5 days) peptide solutions (C, F); of hIAPP(20-29) (A, B, C) and rIAPP(20-31) (D, E, F).

**Supplementary Figure S3.** CD spectra of Aβ peptides (A) Aβ(1-11), (B) Aβ(12-22), (C) Aβ(23-33) and (D) Aβ(34-42) in phosphate buffer, glycerol and TMAO at 0h.

**Supplementary Figure S4.** CD spectra of polyQ peptides (A) Q3, (B) Q6, (C) Q10, (D) Q14, (E) Q20 and (F) Q44 in phosphate buffer, glycerol and TMAO at 0h. (The spectra polyQ at 0h in phosphate buffer was reproduced from our previously published results from Inayathullah et al., 2016 [http://dx.doi.org/10.1016/j.npep.2016.01.011] in order to see the osmolytes effect)

**Supplementary Figure S5.** CD spectra of IAPP; (A) hIAPP(20-29) and (b) rIAPP(20-31) in phosphate buffer, glycerol and TMAO at 0h.

**Supplementary Figure S1**





**Supplementary Figure S2**





**Supplementary Figure S3**


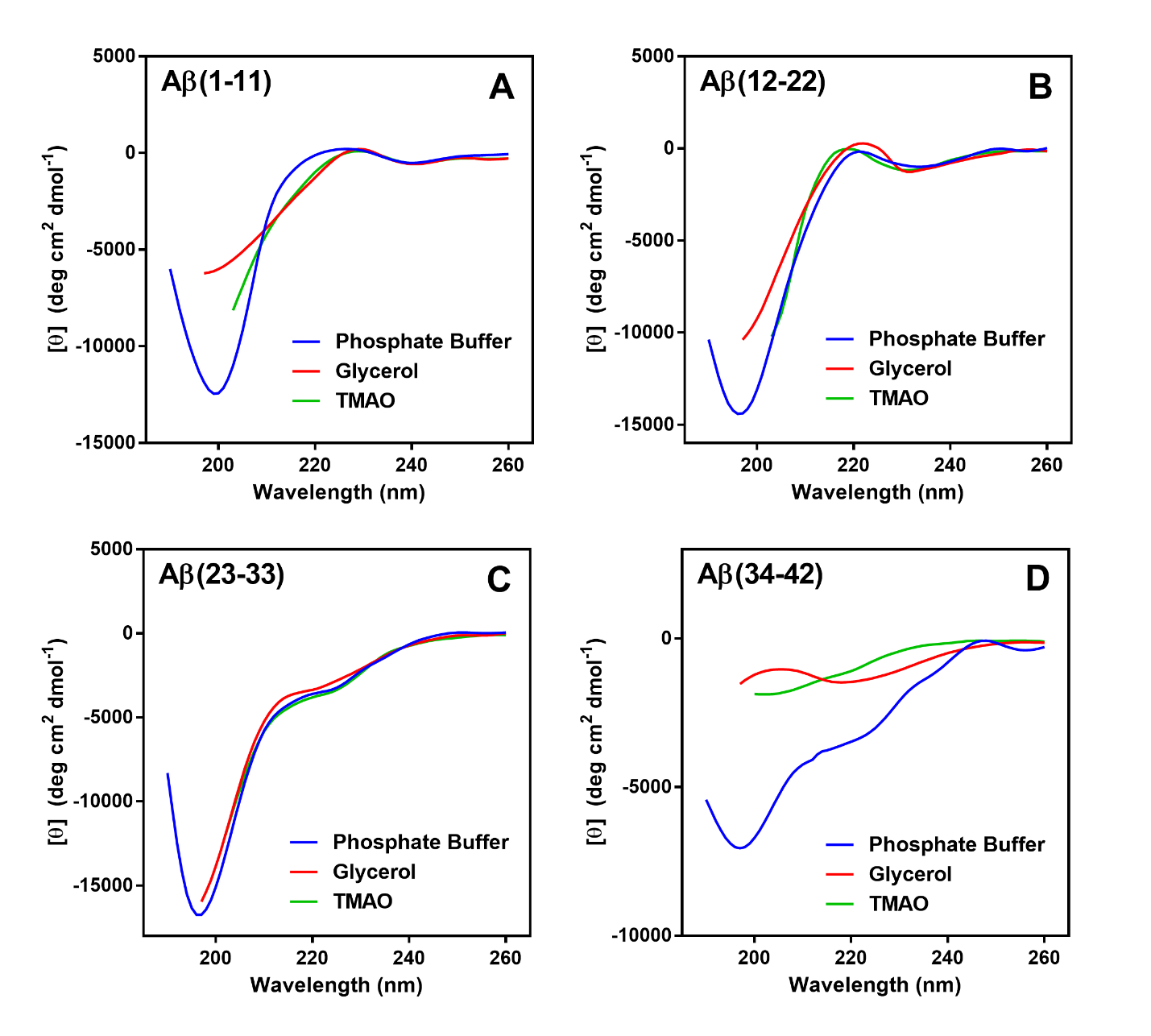


**Supplementary Figure S4**


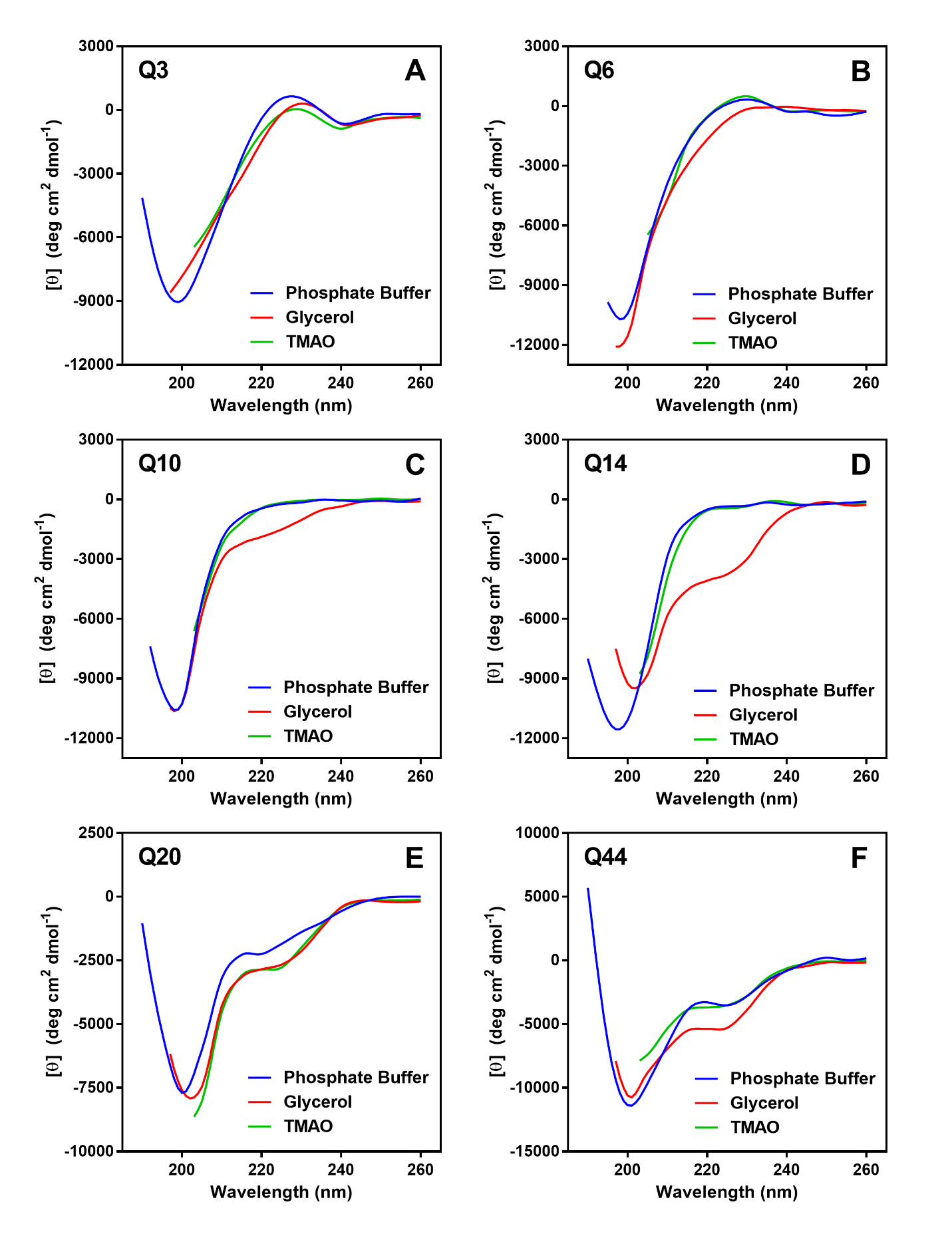


**Supplementary Figure S4**


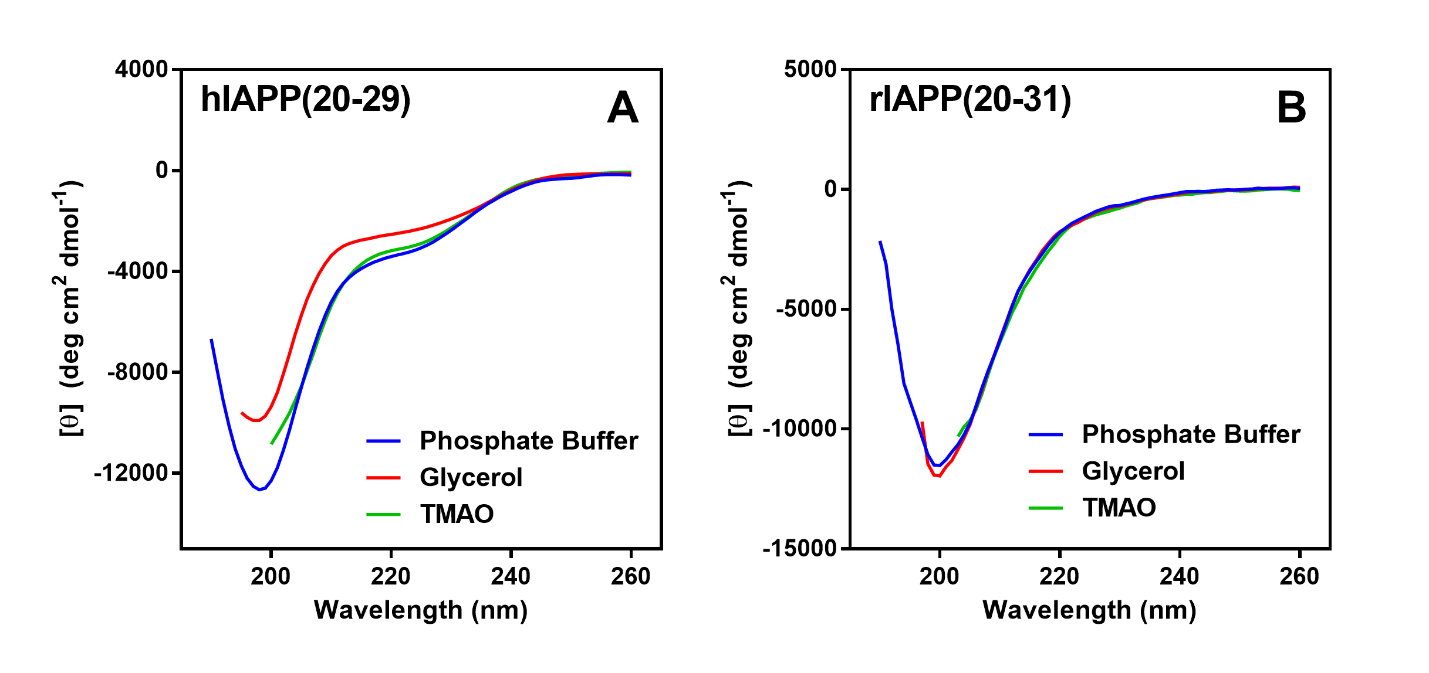

Supplement: Supplementary file 2 — Supplementary material [file mmc2.docx]
